# Effect of *Pistacia atlantica* kernel oil on the quality characteristics of mayonnaise during the storage period

**DOI:** 10.1002/fsn3.4353

**Published:** 2024-08-19

**Authors:** Sara Norouzzadeh, Milad Ghasemzadeh, Hamid‐Reza Akhavan, Khavar Adhami

**Affiliations:** ^1^ Department of Food Science and Technology, Faculty of Agriculture Shahid Bahonar University of Kerman Kerman Iran

**Keywords:** mayonnaise, natural antioxidant, *Pistacia atlantica* oil, sensory attributes, stability

## Abstract

Mayonnaise is an oil‐in‐water emulsion with 65–85% oil, and its physicochemical and sensory characteristics are greatly influenced by the type of oil used. The nutritional value and oxidation resistance of *Pistacia atlantica* oil (PAO) are very desirable due to the essential fatty acids (oleic and linoleic) and antioxidant compounds (phenolics and tocopherols). In this research, the physicochemical and sensory characteristics of mayonnaise sauce (MNS) with different levels of PAO (15% (MNS‐PAO15%), 30% (MNS‐PAO30%), and 100% (MNS‐PAO), as a substitute for soybean oil (SBO)) were compared with sauces containing SBO with antioxidants (MNS‐SBOA) or without antioxidants (MNS‐SBO) during storage for 30 days at 25°C. The results showed that MNS‐PAO and MNS‐SBO exhibited the lowest physical and thermal stability. The MNS‐PAO had the lowest values in peroxide (2.18 meq/kg oil) and thiobarbituric acid (0.21 mg MDA/kg oil) at the end of the storage period. Increasing the PAO level in the mayonnaise formula increased the b* value, but a decreasing trend of b* in these samples was observed during the storage period. The panelists gave the lowest sensory score to the MNS‐PAO sample, but they did not consider a significant difference between the samples containing SBO and the sample containing 15% PAO in terms of color, aroma, and overall acceptance. In general, mayonnaise containing 15% PAO was suggested as a desirable sauce in terms of emulsion and oxidation stability and sensory characteristics.

## Graphical abstract

The study compared mayonnaise sauces with different levels of *Pistacia atlantica* oil (PAO) as a substitute for soybean oil, analyzing their physicochemical and sensory characteristics during a 30‐day storage period. Results showed that sauces with PAO had lower physical and thermal stability but exhibited desirable oxidation resistance with low peroxide and thiobarbituric acid values. The sample with 15% PAO was recommended for its emulsion and oxidation stability, along with its favorable sensory characteristics.

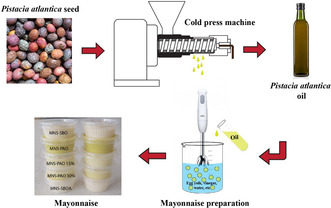

## 1 INTRODUCTION

Mayonnaise is one of the popular condiments that is added to various foods to improve the taste, and it is usually made by mixing vegetable oil, egg (whole or yolk), vinegar, water, and spices (Raikos et al., 2016). Mayonnaise is considered as an oil‐in‐water emulsion with a low pH and high oil content, whose oil content can vary from 65% to 85% depending on the formulation (Di Mattia et al., 2015). Vegetable oils like canola oil, soybean oil, cottonseed oil, and sunflower oil are commonly used in producing mayonnaise. Oil is crucial for determining the viscosity, texture, lubrication, appearance, taste, and shelf life of mayonnaise. The significant amount of oil used to ensure the quality and stability of mayonnaise can be associated with certain cardiovascular diseases and health problems (Muhialdin et al., 2019). Like other high‐fat foods, mayonnaise is prone to spoilage due to auto‐oxidation, which is an important factor in reducing the sensory and nutritional quality of this product during long‐term storage (Li et al., 2014). Therefore, the use of unsaturated oils with higher resistance to oxidation and rich in antioxidant compounds can be effective in improving the quality characteristics of mayonnaise. In this regard, the use of PAO in the formulation of mayonnaise sauce was considered.

Pistacia species from the *Anacardiaceae* family include about 15 species, of which *P. atlantica*, *P. vera*, and *P. khinjuk* grow wild in Iran (Hazrati et al., 2020). The oil content of wild pistachio (*P. atlantica*) is almost similar to that of pistachio (*Pistacia vera* L.) and is about 55–58%. Also, the kernel of *P. atlantica* fruit has a higher oil content compared to other vegetable oils such as sunflower, peanut, cottonseed, and corn (Tavakoli & Khodaparast, 2008). In Iran, the wild pistachio fruit, which is called Beneh, is ground and mixed with other ingredients to be consumed as a traditional food by local people (Hatamnia et al., 2014). These species are rich in essential unsaturated fatty acids such as oleic acid (52.03%–57.39%) and linoleic acid (12.02%–23.5%), as well as saturated fatty acids like palmitic acid (12.44%–23.4%) (Hazrati et al., 2020; Tavakoli et al., 2015). As a result, this oil can be used for cooking and salad oils or in margarine formulations (Saber‐Tehrani et al., 2013).

Antioxidant compounds in plants play an important role in maintaining human health. Scientific evidence suggests that antioxidants, such as phenolic compounds, reduce the risk of chronic diseases such as cancer and heart disease by scavenging free radicals (Hatamnia et al., 2014). Phenolic compounds act as antioxidants in biological systems and food products by donating electrons and terminating free radical chain reactions (Liu et al., 2007). The most common synthetic antioxidants used to prevent lipid oxidation in food products are butylated hydroxytoluene (BHT), butylated hydroxyanisole (BHA), tert‐butyl hydroquinone (TBHQ), and EDTA. Despite their economic advantages, they often receive a negative perception due to their artificial nature (Ghorbani Gorji et al., 2016). PAO is a rich source of natural antioxidants due to the presence of unique phytosterols and tocopherols (Saber‐Tehrani et al., 2013). Tavakoli et al. (2016) found the total phenolic content of *P. atlantica* and *P. khinjuk* oils as 75.22 and 26.6 mg/kg, respectively. Also, the tocopherol content of *P. atlantica* oil and *P. khinjuk* oils was 845.33 oil and 619.4 mg/kg.

The complete or partial replacement of conventional oils used in the formulation of mayonnaise with other oils has been reported in various studies. In this regard, Muhialdin et al. (2019) reported higher antioxidant activity of mayonnaise samples with complete and partial replacement of soybean oil by virgin coconut oil. The replacement of corn oil with date seed oil in the mayonnaise formula showed that the sensory characteristics of the mayonnaise containing date seed oil were superior compared to the commercial sample containing corn oil (Mohamed, 2011).

The *Pistacia atlantica* tree grows naturally/wild and produces abundant fruit. The oil of this fruit has a very suitable profile of fatty acids and is rich in antioxidant compounds, including phenolic compounds and tocopherols. There are no reports in the literature on the use of PAO in mayonnaise formulation. Continuous research on PAO, especially its use in food formulations, can lead to the development of the use of this oil in the food industry. Therefore, the purpose of this research is to evaluate the effect of replacing SBO with PAO on some quality characteristics of mayonnaise during the storage period.

## 2 MATERIALS AND METHODS

### 2.1 Materials

The *P. atlantica* (Beneh) oil produced by cold pressing (Wissta‐Shahd Co., Kerman) and soybean oil without antioxidants (Golnaz vegetable oil Co., Kerman, Iran) were donated. Other ingredients, including egg yolk, vinegar, sugar, and salt, were prepared at the local market in Kerman. Arabic gum, potassium sorbate, BHT, potassium iodide, 1,1,3,3‐tetraethoxypropane, and thiobarbituric acid were purchased from Merck, Germany.

### 2.2 Preparation of mayonnaise sauce

To prepare mayonnaise samples, salt, sugar, potassium sorbate, and Arabic gum were first dissolved in water, then added to egg yolk and stirred. In the next step, vinegar was added, and the oil was slowly added to the mixture to form a mayonnaise emulsion. Mayonnaise samples were prepared using the same percentage of ingredients (oil (66%), egg yolk (13.25%), vinegar (7.7%), water (7.5%), sugar (3.85%), salt (1.5%), gum arabic (0.2%), and sorbate (0.1%)). The difference between the mayonnaise samples was the type of oils used (PAO and SBO) and the ratio of oils in the formula. Mayonnaises included samples with 15%, 30%, and 100% PAO and MNS‐SBOA (100% soybean oil containing BHT), which were compared with MNS‐SBO as a control (100% soybean oil without antioxidants). The samples were placed in an incubator (Jal Tajhiz, Iran) at 25°C for 30 days. The following physicochemical tests were performed at 1, 10, 20, and 30 days of storage time, while sensory analysis was performed only on the first day.

### 2.3 Measurement of pH and titratable acidity

First, the mayonnaise samples were kept at room temperature, and then the pH of the sauces was measured with a pH meter (Zag Chimi, Iran). The titratable acidity (TA) of samples was evaluated by the AOCS Official Method Te 2a‐64 (AOCS, 2017). For this purpose, 15 g of mayonnaise was mixed with 200 mL of distilled water and then titrated with 0.1 M NaOH, using phenolphthalein as an indicator of the titration end‐point (pale pink color). The TA was expressed in g of acetic acid per 100 g of sample.

### 2.4 Physical and thermal stability evaluation

To evaluate the physical stability of mayonnaise, 50 g of the sample was weighed in the test tube and centrifuged at 2795 × g for 30 min at room temperature (Sigma 2‐16P, Germany). Then, the upper phase (oil layer) was removed, and sediment volume was measured. The physical stability of mayonnaises was calculated using the following equation (Mousakhani‐Ganjeh & Goli, 2021):

$$\text{Physical stability of the emulsion}=\frac{\text{Volume of the remaining emulsion}}{\;\text{Total volume}}\times 100$$

To evaluate the thermal stability of mayonnaise, 50 g of the samples were weighed in the test tube and placed in a water bath (Memmert, Germany) at 80°C for 30 min, and then immediately cooled. The samples were centrifuged at 2795 × g for 30 min at room temperature. After removing the upper phase (oil layer), sediment volume was measured. The thermal stability of mayonnaises was calculated using the following equation (Mousakhani‐Ganjeh & Goli, 2021):

$$\text{Thermal stability emulsion}=\frac{\text{Final emulsion volume}}{\text{The volume of the primary emulsion}}\times 100$$

### 2.5 Measurement of peroxide value (PV) and thiobarbituric acid (TBA)

The mayonnaise samples were first frozen at −80°C for 48 h to extract the oil from the emulsion. The frozen samples were placed at 4°C for 2 h to break the emulsion and then centrifuged at 2795 × g for 20 min (Hakimian et al., 2022).

To measure the PV, 5 g of the extracted oil was dissolved in 30 mL of acetic acid (0.1 M)/chloroform (3:2 v/v) and 0.5 mL of saturated potassium iodide solution was added. 30 mL of distilled water was added immediately, and 3 drops of starch solution (10% w/v) were used as the endpoint indicator. Sample and control solutions were titrated with 0.1 N sodium thiosulfate until they became colorless. The peroxide value was calculated based on the equation PV = [(*S* − *B*) × *N* × 1000]/*W* (Muhialdin et al., 2019), where *S* and *B* are the volumes (mL) of sodium thiosulfate titrant used in the titration of the lipid sample and blank, respectively. *N* is the normality of the titrant, and *W* is the weight (g) of the lipid sample.

The TBA value was measured based on the AOCS Cd 19–90 method (Hakimian et al., 2022). 0.2 g of the extracted oil phase was diluted with 1‐butanol in a 25‐mL volumetric flask. A mixture of 5 mL of this solution and 5 mL of TBA (0.2% w/v) reagent was combined, heated in a water bath at 95°C for 2 h, and rapidly cooled in an ice bath. The absorbance of the samples was measured at 532 nm against a corresponding blank with a UV/Vis spectrophotometer (Unico 2802, China). A standard curve was prepared by plotting absorbance values versus the different concentrations of 1,1,3,3‐tetraethoxypropane (0.1–0.5 ppm). The results were expressed as milligrams of malondialdehyde per kilogram of mayonnaise oil (mg MDA/kg oil).

### 2.6 Measurement of total phenol content

The measurement of total phenolic content (TPC) was carried out using the method of Fuentes et al. (2012), with slight modifications. For the extraction of phenolic compounds, the extracted mayonnaise oil (2.5 g) was dissolved in 5 mL hexane and mixed with 3 mL of methanol/water (40:60, v/v) and then vortexed for 2 min. The hexane phase was separated by centrifugation (1370 × g for 10 min). The extraction was repeated with 3 mL methanol/water (40:60, v/v) and the methanolic phases were combined. The methanolic phase (0.2 mL) was diluted with 2.5 mL of water, then 0.25 mL of Folin–Ciocalteu reagent and 0.5 mL of sodium carbonate (7.5% w/v) were added, and finally diluted to 5 mL with distilled water. Similarly, control and gallic acid standards (10–250 mg/L) were also prepared. The samples were stored for 2 h in a dark place, and the absorbance was measured by a UV/Vis spectrophotometer at a wavelength of 725 nm. Based on the calibration curve, the TPC of the samples was expressed as mg of gallic acid equivalents (GAE)/L oil.

### 2.7 Measurement of color indices

The color indices (L*, a*, and b*) of mayonnaise samples during the storage period were measured using a TES‐135A colorimeter (Taiwan). First, the colorimeter was calibrated with a standard white surface (L* = 96.26, a* = −0.680, b* = 0.966), and then the L* (brightness), b* (yellow‐blue), a* (red‐green), and the total color difference (∆*E* = ((*L*_0_ − L*)^2^ + (*a*_0_ − a*)^2^ + (*b*_0_ − b*)^2^)^1/2^) of samples were measured (Alizadeh, Mortezapour, et al., 2019).

### 2.8 Sensory evaluation

Eight trained panelists (five men and three women) with an average age of 20–45 years were selected among students and staff of the Food Science and Technology department. The panelists were familiar with the quality characteristics of mayonnaise sauce and evaluated the sensory attributes (color, texture, taste, aroma, and overall acceptance) of samples with a 5‐point hedonic method (very poor (1) and very good (5)) on the first day of storage. Containers of lettuce salad mixed with desired mayonnaises were given to each panelist to record the scores in the sensory form. The panelists used water and unsalted bread for palate cleansing between the samples.

### 2.9 Statistical analysis

This experiment was conducted as a factorial based on a completely randomized design with 3 repetitions. Statistical tests were performed with one‐way analysis of variance (ANOVA) and Duncan's multi‐range test at a significant level (*p* < .05) by SAS software. The results were expressed as mean ± SD.

## 3 RESULTS AND DISCUSSION

### 3.1 pH and titratable acidity

**FIGURE 1:**
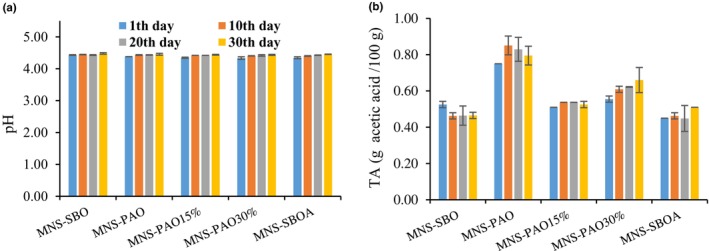
The variation of pH and TA in different mayonnaise formulations containing PAO and SBO during 30 days of storage at 25°C. Data are presented as mean ± SD.

The pH of the samples was significantly affected by the oil type and storage time (*p* < .05). As the storage time increased, the pH of all samples increased. However, the pH of the samples was in the range of 4.30–4.50 during storage (Figure 1a). The results showed that the storage time had no significant effect on the TA of the samples (*p* > .05), and only the oil type used in the mayonnaise formula showed a significant effect (*p* < .05) on the TA (Figure 1b). The highest values of TA (0.75–0.85%) were observed in the MNS‐PAO. The acetic acid content of all sauce samples was similar; therefore, the difference in TA seems to be mainly due to the free fatty acids (FFA) of the used oil in the MNS formula. The initial FFA values of PAO and SBO were 1.36 and 0.08 mg oleic acid/100 g oil, respectively. The TA value of MNS is related to the added acetic acid and the free fatty acids inherent in the oil or the result of the possible hydrolysis of triacylglycerols in the presence of water or oxidation reactions (Alizadeh, Abdolmaleki, et al., 2019; Kishk & Elsheshetawy, 2013). Based on the results, higher TA values were assigned to the sample prepared with PAO because PAO naturally contains a significant amount of free fatty acids (Zarei Jelyani et al., 2021). As a result, the MNS‐PAO sample had the highest acid value compared to other treatments.

### 3.2 Physical and thermal stability of the emulsion

**FIGURE 2:**
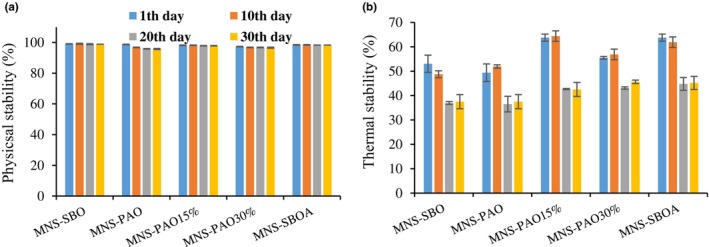
The variation of the physical and thermal stabilities of different mayonnaise formulations containing PAO and SBO during 30 days of storage at 25°C. Data are presented as mean ± SD.

The results showed that the effects of storage time and oil type and their interaction effects on the physical and thermal stability of the MNS emulsions were significant (*p* < .05). The physical and thermal stabilities (Figure 2a,b) of the samples were significantly decreased at the end of the storage period compared to the first day (*p* < .05). The lowest level of physical stability was observed in the MNS‐PAO. In other words, the physical stability was increased with the reduction of the PAO ratio in the mayonnaise formula. The physical stability of the MNS samples containing PAO decreased by 3% during the storage periods, while in other samples, the rate of change was less than 1%. The highest thermal stability (63.75%) was related to the MNS‐PAO15% and control, and the lowest level (49.38%) was related to the MNS‐PAO on the first day of the storage period. The thermal stability of MNS‐SBO and MNS‐PAO decreased to ~37.5% at the end of the storage period, which was the lowest stability among the treatments.

The composition of fatty acids and their degree of unsaturation in triacylglycerols vary due to the oil source and oil processing and modification methods. As a result, they can affect the emulsification process and the stability of the resulting emulsion during the storage period (Gmach et al., 2019). The higher degree of unsaturation of the oil used in the sauce formula leads to maintaining the stability of MNS during the long‐term storage period (Saberi & Mohammadifar, 2012). It should be noted that the oleic acid and linoleic acid contents of PAO are ~51 and 29%, and those of soybean oil are ~23 and 54%, respectively (Saberi & Mohammadifar, 2012; Saber‐Tehrani et al., 2013). Therefore, the higher degree of unsaturation of soybean oil led to the greater stability of MNS‐SBO compared to MNS‐PAO.

In general, the stability of the emulsion indicates the resistance of the emulsion against the change of physicochemical properties during the storage time. Food emulsions can become unstable due to various physicochemical mechanisms such as gravitational separation, flocculation, coalescence, partial coalescence, Ostwald ripening, and phase inversion (Kantekin‐Erdogan et al., 2019).

### 3.3 Oxidative stability

**FIGURE 3:**
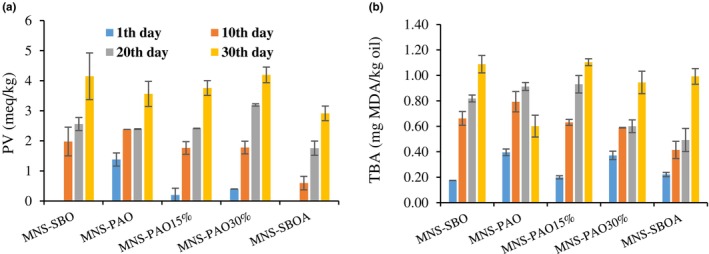
The variation of PV and TBA of different mayonnaise formulations containing PAO and SBO during 30 days of storage at 25°C. Data are presented as mean ± SD.

The effect of oil type and storage time and their interaction on the PV and TBA were significant (*p* < .05). The PV and TBA of the samples increased significantly (*p* < .05) during the storage period (Figure 3a,b). Mayonnaise, like some food products, has a high content of unsaturated oil, and as a result, it is susceptible to oxidative deterioration through the autoxidation of unsaturated fatty acids (Depree & Savage, 2001). During the primary stage of lipid oxidation, hydroperoxides are produced, and the amount of these compounds is determined by the PV (Khalid et al., 2021). The primary products of lipid oxidation are very unstable and decompose into various secondary products, such as alkanes, ketones, and aldehydes. Malondialdehyde is one of the dominant aldehydes produced in the secondary oxidation stage and is measured by the TBA (Luan et al., 2023).

In terms of the oxidative stability of mayonnaise, the exposure of a large surface of the oil phase to the aqueous phase increases the possibility of lipid oxidation reactions due to the dissolved oxygen in the aqueous phase of the emulsion (Depree & Savage, 2001). Based on the results, the highest amounts of PV (1.38 meq/kg oil) and TBA (0.40 mg MDA/kg oil) were observed in the MNS‐PAO on the first day of the storage period. This issue can be attributed to the quality of PAO, especially its high initial acidity. Because the *Pistacia atlantica* tree grows wild in some mountainous areas of Iran, there is no proper control over the growing conditions and postharvest of the *Pistacia atlantica* fruit. The PV and TBA of the MNS‐SBO were respectively 4.15 meq/kg and 0.91 ppm at the end of the storage period, which were significantly higher than other treatments. But the MNS‐PAO had the lowest PV (2.18 meq/kg oil) and TBA (0.21 mg MDA/kg oil) at the end of the storage period. The reason can be attributed to the direct reaction of various and natural antioxidants (such as tocopherols and phenolic compounds) in the PAO with free radicals formed in the initial stages of oxidation, which lead to the scavenging of radicals from food products (Shahin et al., 2014). In this regard, the results of the present study were consistent with previous studies. Özdemir et al. (2018) reported that the use of black cumin oil in the production of mayonnaise increased the oxidative stability of mayonnaise due to the presence of natural phenolic compounds and their antioxidant effects. Also, the use of grape seed extract in the mayonnaise formula reduced the rate of lipid oxidation (Altunkaya et al., 2013).

### 3.4 Total phenolic content

**FIGURE 4:**
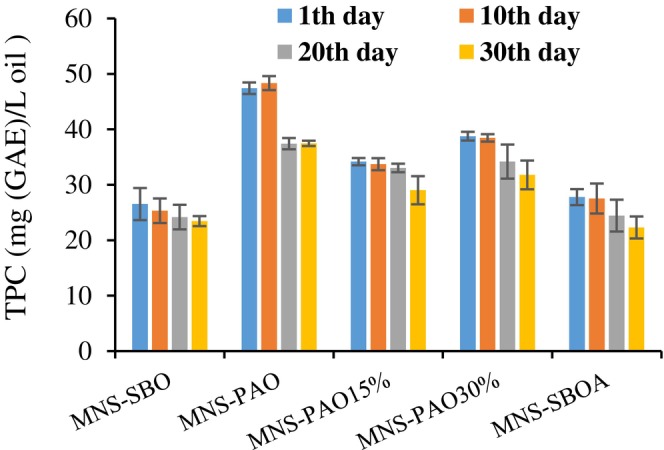
The variation of TPC of different mayonnaise formulations containing PAO and SBO during 30 days of storage at 25°C. Data are presented as mean ± SD.

The results showed that the effects of oil type and storage time on the TPC of MNS samples were significant (p < .05). The highest content of TPC was observed in the MNS containing PAO (Figure 4), which is the result of the high content of phenolic compounds in PAO (Tavakoli et al., 2016).

The TPC of the samples decreased from 12 to 21% during storage for 30 days at 25°C. The highest decreasing trend of TPC (21%) was observed in the MNS‐PAO, but this sample showed the highest oxidation stability. It should be noted that there is a direct correlation between the content of phenolic compounds and their free radical scavenging capacity. Therefore, the decreasing trend of phenolic compounds during the storage of mayonnaise can be attributed to the antioxidant activity of these compounds in preventing the primary oxidation of lipids (Tavakoli et al., 2019). Phenolic compounds prevent or delay the formation of primary and secondary oxidation products in mayonnaise by three mechanisms: (1) breaking oxidation chain reactions, (2) destroying hydroperoxide, and (3) chelating metal ions (Altunkaya et al., 2013). On the other hand, these compounds act as strong hydrogen donors and play an important role in scavenging free radicals (Hatamnia et al., 2014). For this reason, the presence of these compounds in PAO led to fewer changes in the PV and TBA of the MNS containing PAO compared to the MNS containing SBO.

### 3.5 Color evaluation

**FIGURE 5:**
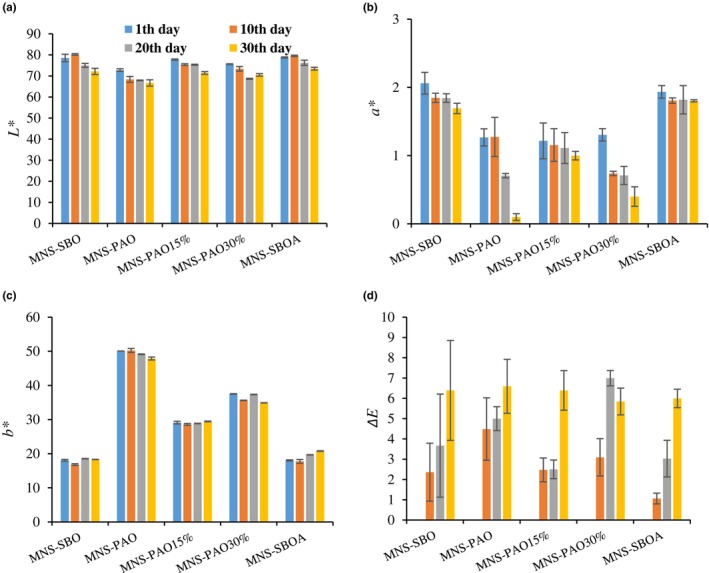
The variation of color indices (L*, a*, b*, and Δ*E*) of different mayonnaise formulations containing PAO and SBO during 30 days of storage at 25°C. Data are presented as mean ± SD.

Based on the results (Figure 5a–d), the effects of oil type and storage time on the color indices of brightness (L*), redness (a*), yellowness (b*), and Δ*E* of the mayonnaises were significant (*p* < .05). The L* of MNS has a certain effect on the perception of the appearance of the product (Kumar et al., 2021). As shown in Figure 5a, the L* of the samples decreased significantly during the storage period (*p* < .05). This trend is probably due to droplet size changes in the dispersed phase, which causes more light absorption of the sauce ingredients and less light reflection (Santipanichwong & Suphantharika, 2007). The Δ*E* of the samples increased significantly (*p* < .05) with increasing storage time (Figure 5d). The decrease in L* had the greatest effect on *ΔE* values. Also, the b* of the samples was affected by the type of oil, so on the first day, the b* values of MNS samples containing SBO and PAO were around 18 and 50, respectively. The value of b* in the MNS containing PAO was higher than other samples, which can be related to the presence of certain pigments, such as carotenoids. In this regard, the considerable content of carotenoid and chlorophyll pigments in PAO has been reported by Saberi and Mohammadifar (2012). In this regard, with the increase in PAO ratio in the MNS formula, the b* values increased (Figure 5c). The decreasing trend of b* values was observed in these samples during the storage period, which could be caused by the degradation of pigments, including carotenes (Santipanichwong & Suphantharika, 2007). Nikkhah et al. (2021) reported that the replacement of cream fat with PAO at the level of 10% led to a decrease in L* and an increase in the b* of the cream, which was consistent with the results of the present study.

### 3.6 Sensory evaluation

**FIGURE 6:**
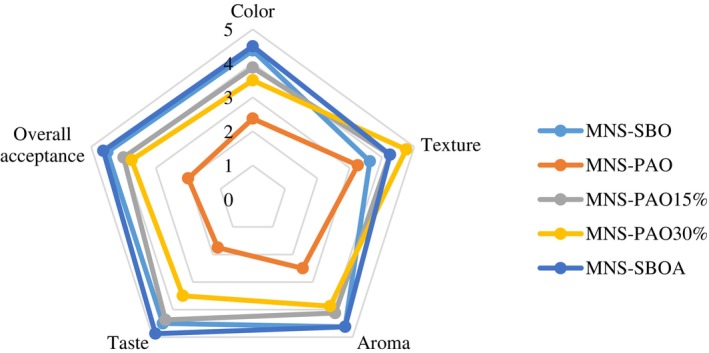
Sensory attributes (color, taste, aroma, texture, and overall acceptance) of the mayonnaise sample containing PAO and SBO on the first day of storage.

The sensory results of the samples prepared with the two types of oils are shown in Figure 6. There were significant differences in terms of taste, color, aroma, texture, and overall acceptance between different samples, which indicates the effect of oil type on the sensory characteristics of mayonnaise (*p* < .05). The results showed that the MNS‐PAO obtained the lowest score of sensory attributes, but the panelists did not observe a significant difference between the MNS‐SBO and MNS‐PAO15% in terms of color, aroma, and overall acceptance. The MNS‐SBO and MNS‐PAO samples obtained the highest and lowest color scores, respectively. The yellowness of MNS‐PAO was higher than the other samples, which were given a lower score by the panelists due to the significant difference in the color of this sample from the commercial samples. In addition, the aroma of the MNS‐PAO received the lowest score due to the specific aroma of cold press PAO. Safiaghdam et al. (2021) reported a similar result with mayonnaise containing a high level of peppermint essential oil and attributed the decrease in the desirability of the samples to the strong aroma and taste of the peppermint essential oil. The highest sensory scores of tastes (4.4–4.9) were observed in MNS‐SBO, MNS‐SBOA, and MNS‐PAO15% samples. An increase in the ratio of PAO in the MNS formula led to a decrease in the score of taste, so the MNS‐PAO received a score of 1.75. The decrease in taste acceptance can be attributed to the specific phenolic compounds of PAO, which cause a strong pungent taste in this oil. Similarly, Di Mattia et al. (2015) attributed the increased spicy flavor of mayonnaise containing extra virgin olive oil to its high phenolic content, which reduced its desirability. Interestingly, the texture of the MNS‐PAO30% obtained the highest score (4.75). In general, increasing the ratio of PAO decreased the scores of the sensory attributes in the MNS samples. Nikkhah et al. (2021) reported a decrease in the sensory acceptance of cream with an increase in the PAO ratio in low‐fat cream formulas. The complete replacement of SBO with PAO in the low‐fat cream reduced the overall acceptance scores from 4.63 to 2, which is consistent with the results of the present study. The overall acceptance score of the MNS sample containing virgin coconut oil was also lower than the sample prepared with SBO (Muhialdin et al., 2019).

## 4 CONCLUSION

In this research, the effect of complete or partial replacement of SBO with PAO on the quality characteristics of MNS formulas was investigated. Based on the results, the TPC of the MNS samples increased with the increase in the ratio of PAO, which led to an increase in the oxidative stability of the samples, but it decreased the emulsion stability of the MNS samples during the storage period. The sensory evaluation revealed that increasing the ratio of PAO in the sauce formulas reduced the scores of the sensory attributes. In general, the samples containing 15% PAO received higher sensory scores and had good oxidative and physical stability during the storage period. As a result, it can be considered as a desirable option in the food industry, considering the nutritional challenges of synthetic antioxidants. It seems that the only challenge of pure cold‐pressed PAO is its high content of free fatty acids. Therefore, it is possible to increase the quality of PAO by controlling the post‐harvest conditions of *Pistacia atlantica* fruit and improving its oil extraction methods.

## AUTHOR CONTRIBUTIONS

**Sara Norouzzadeh:** Data curation (equal); investigation (equal); writing – original draft (equal). **Milad Ghasemzadeh:** Conceptualization (equal); data curation (equal); writing – original draft (equal). **Hamid‐Reza Akhavan:** Methodology (equal); supervision (lead); writing – review and editing (lead). **Khavar Adhami:** Data curation (equal); methodology (equal).

## FUNDING INFORMATION

This work was supported by Shahid Bahonar University of Kerman.

## CONFLICT OF INTEREST STATEMENT

The authors declare no conflict of interest.

## ETHICS STATEMENT

The manuscript has no ethical issue.

## CONSENT FOR PUBLICATION

All authors agreed to the publication of this manuscript.

## Data Availability

The data that support the findings of this study are available on request from the corresponding author.
